# Comparative reflections of Australian and African female academics on working from home during COVID-19

**DOI:** 10.3389/fpsyg.2022.944384

**Published:** 2022-08-22

**Authors:** Upasana G. Singh, Rashmi Watson, Chenicheri S. Nair

**Affiliations:** ^1^Department of Information Systems and Technology, University of KwaZulu-Natal, Durban, South Africa; ^2^Medical School, University of Western Australia, Perth, WA, Australia; ^3^Victorian Institute of Technology, Melbourne, VIC, Australia

**Keywords:** female academics, Australia, Africa, work from home, COVID-19, pandemic

## Abstract

The COVID-19 pandemic has taken a heavy toll on women globally, and female academics were no exception to the unprecedented, forced shift to working from home. Increased workloads, additional domestic responsibilities, and extended working hours have led to high levels of dissatisfaction among this group of academics. This disruption has also impacted mental and physical wellbeing. There has been limited research on the experiences of female academics during the transition to the new work environment in the early stages of the pandemic. This research compares the opportunities and challenges faced, as well as the support received, by female academics in Australia and Africa. Specifically, this study reports on the changing roles; demands of increased workloads; challenges, and opportunities faced both personally, and in general, an exploratory, qualitative approach was adopted in this study. An online questionnaire was developed and distributed through mailing lists in Africa and Australia; LinkedIn; as well as a personal invitation by the researchers on WhatsApp and email. Purposeful and snowballing sampling female academics in Australia and Africa were targeted, Inclusion criteria for this study were female academics employed at any higher education institution (HEI), private or public, in contract, and part-time and full-time employment in Australia and Africa since the start of the pandemic (February 2020). A total of 171 respondents (144 from Australia and 27 from Africa) were received from a larger, global study with 260 responses gathering data about female academics’ experiences during COVID-19. The data were analyzed using thematic and inductive analyses. The study sheds light on workload, motivation, perceptions about career progression, and work status. The research contributes to the body of knowledge of femaleacademic work, gender disparity, and higher education impact during COVID-19. The research aims to add value to the literature that supports the growing feminism in academia to ensure HEIs support this cohort of academics.

## Introduction and background

Work–life balance of women employees is an integral issue in the employment world globally. There is ample evidence in the academic literature which shows the issues, benefits and need for policies to help women in their work environment (Mani, 2013; Ravi and Anulakshmi, 2021). However, although these factors are well known, little has been researched with the recent disruption due to the COVID-19 pandemic on the effects on female academics. This study aims to compare the opportunities and challenges of female academics in Australia and Africa during this period of disruption so as to understand the female academics workloads and related issues when the world of higher education was forced to deliver programs digitally mainly from their homes. Research by Lunyolo et al. (2014) reports that socio–cultural barriers hinder women not only in academia but also in other areas. Further, Morley (2013) explains that the culture in academia is frequently viewed as unfriendly and unaccommodating to women. In Africa, Nomadolo’s (2017) work shows that the gender stereotypes and prejudices are exhibited by a number of organizations with males classed as privileged. Although gender equality in Africa and Australia are at different stages, some countries in Africa have policies in place to ensure gender equality, e.g., South Africa, although women remain marginalized (The Conversation, 2022). However, Australia is much advanced; for example, with the Australian Equal Opportunity law promoting women in the workplace.

## Literature review

### Working from home: The balancing act

The rapid transition almost overnight for many from working at a desk in an office to working from home (WFH) has been reported to have many positive benefits. These include increased flexibility (Mattey et al., 2020; Watson et al., 2022a) which may have led to the reported decreased burnout (Hoffman et al., 2020), increased work–life balance satisfaction (Dockery and Bawa, 2020), and where many stated they could sustain WFH indefinitely (Watson et al., 2022a). The remote work has shown to strengthen employee and employer relationships through its support of employee wellbeing (Shirmohammadi et al., 2022). The nature of work and women’s roles in the pandemic period has shown that women have adapted in numerous ways such as sacrificing work and non-work roles and boundaries have been managed through structural support such as flexibility and social support such as empathy (Kossek et al., 2021; Wethal et al., 2022). The term “boundary-work” has been used by Wethal et al. (2022) to describe the changes that have been made to recalibrate work and home duties and can also be referred to as “boundary traffic” as a congestion between home and work.

The shift to WFH has shown many benefits and positive outcomes, many issues in work–life balance have also been reported. These include increased work in traditional gendered role responsibilities, blurred delineation between home and work lives and increased depression, exhaustion and burnout (De Vos, 2020; Bezak et al., 2022). Female academics, in particular, are managing multiple and demanding roles in their home environment (household work, children, extended families, home schooling, and teaching and research demands) pre- and during COVID-19 (Günçavdi et al., 2017; Madgavkar et al., 2020; Parlak et al., 2021; Bezak et al., 2022). It is reported that in the Middle East and in North Africa, it is as high as 80–90% (Madgavkar et al., 2020).

## Gendered issues

Females have been impacted during COVID-19 with increased care roles, increased household responsibilities, being able to manage home schooling while managing their own workloads (Feng and Savani, 2020; Mattey et al., 2020). High-powered women balance professional and home lives “spectacularly” (Rottenberg, 2014, p. 248). Ethnicity and cultural context play a role in barriers to how well female academics have been able to manage work and life balance where those of African/Black females stated greater issues in cost of communication, connectivity, social isolation and a high burden of care responsibilities with external family members (Leal Filho et al., 2021; Bezak et al., 2022).

Greater gender gaps have now been reported since COVID-19 with perceived job satisfaction (Feng and Savani, 2020) and distractions and disturbance from family (Watson et al., 2022a) and decreased motivation toward career progression (Watson et al., 2022b). The current pandemic has deepened the inequalities among genders and is an issue that needs attention and adequate response by higher education institutions (Parlak et al., 2021; Watson et al., 2022b). Madgavkar et al. (2020) are pushing the key message that the sooner the policy makers and leaders act on gender equality as COVID continues, the greater the benefits for economic growth.

## Academic transition to online teaching and learning

Online teaching and learning have now become the “new norm,” the way academics and HEIs have had to adapt in a short space regardless of their academic preparedness (Bezak et al., 2022). Digital technologies have taken the interest of HEIs due to its ability to manage the high volumes of in-person teaching, the most commonly used mode of teaching prior to the impact of COVID-19. Academics have certainly transformed themselves in their understanding of online pedagogy (Daniela, 2019) and have been thrust into learning about digital technologies, academic online integrity, (Singh, 2021) and managing online engagement with their students in both synchronous and asynchronous modes. Evidence since the start of COVID-19 supports the need for academics to create an engaging online environment to encourage student interaction, communication, motivation, and learner achievement; all of these require a great amount of effort to accomplish (Oliver et al., 2014), especially if it is a new mode of teaching.

The support mechanisms for academic staff thus have become a critical element of HEI importance during this pandemic. Adequate support and resourcing are critical at classroom level for pedagogical support, quality professional development, academic integrity of teaching and assessment practices and at institutional level including adequate resourcing, technological infrastructure (Stone and Springer, 2019). Numerous online tools and technologies have been applied with the learning management system (LMS) being one of the most common platforms across HEIs for information management, communication, and assessment upload. Regardless of the digital technologies, if academics are not properly supported through their online methods, then the students can be passive learners who are disengaged with academic staff, their peers, and with content (Oliver et al., 2014; Martin et al., 2019). Online engagement, just as in face-to-face engagement remains a key component of maintaining quality pedagogy and assessment practices (Martin et al., 2019).

## Methodology

The research was led through Australia where the ethics approval was received through the University of Western Australia (REF: ET000781). Due to COVID-19 social distancing and with the intention to capture a broad scope of global responses, an online survey was developed to collect data from the female academics across the world, using a mixed-method approach with both open and closed questions. There were 33 questions in total with 24 being closed questions including demographics, and 9 open-ended questions, asking specific perceptions about the academic workload (teaching and research), career progression, support, motivation, and academic leadership. The specific questions being reported from the larger online survey are s follows:

1. What are some of the changes to the academic role being experienced during COVID-19 (workload, ease of managing from home)?
2. What are some of the opportunities being perceived by female academics as a result of the changing roles during COVID-19?

The key method used to gather responses came from email invitations from each of the researchers, email lists from their universities (permission as per ethics protocols), social media including LinkedIn, Twitter, Facebook, and WhatsApp, and the researchers’ own academic networks. The data were collected from July 2021 to October 2021 with the participants spanning across the globe to seek participation. This study explores a comparison between the Australian and African respondents as this is where the researchers are based.

The data were analyzed by applying thematic analysis for the open-ended questions and descriptive statistical analysis for all closed questions. Thematic analysis is a commonly used tool in qualitative research adopted to identify, analyze, describe, organize, and report themes found within a data set (Nowell et al., 2017; Terry et al., 2017; Kiger and Varpio, 2020). The specific thematic applied was inductive thematic analysis, often used in mixed-method designs as the theoretical flexibility of thematic analysis makes it a more straightforward choice than approaches with specific embedded theoretical assumptions (Creswell, 2003). An inductive thematic approach allows research findings to emerge from frequent, dominant, or significant themes inherent in raw data as used in grounded theory (Braun and Clarke, 2006; Liu, 2016).

## Results

There were 260 responses from female academics across the globe (55% were from Australian academics and 10% from African academics). A total of 171 respondents (144 from Australia and 27 from Africa) were included in this study from the larger, global study gathering data about female academics’ experiences during COVID-19.

### Role changes (due to COVID-19)

As listed in Table 1, the majority of respondents claimed their workload *had increased* since the start of COVID-19 in varying degrees. The Australian cohort was (85%) agreement and the African respondents were (89%). Most respondents claimed that their workload had increased “a great deal”; Australia (43%) and Africa (48%) followed by “increased somewhat.” A smaller (10%) of the Australian respondents stated that the workload had stayed approximately the same and (7%) from the African respondents. The lowest response was in relation to the “workload decreasing” in overall responses, with the Australian cohort (4%) and African cohort (3%).

**TABLE 1:** Workload changes since the start of the pandemic.

Workload changes	Increased a great deal *N* (%)	Increased somewhat	Increased at the start of COVID-19, but has settled back to the normal pattern	Stayed approximately the same	Decreased somewhat	Decreased a great deal
Australia	62 (43%)	43 (30%)	17 (12%)	14 (10%)	2 (1%)	4 (3%)
Africa	13 (48%)	9 (33%)	2 (7%)	2 (7%)	1 (3%)	0 (0%)

In response to having to work remotely, the responses were 100% for both cohorts of respondents; Australia = 144 (100%) and Africa = 27 (100%).

### Managing working from home with others

As reported in Table 2, the majority of respondents in both cohorts stated it was “easy” managing working from home with others, which could have included children, partners, parents, friends, or co-residents.

**TABLE 2:** Managing working from home with others.

	Difficult	Easy
Australian	57 (40%)	87 (60%)
African	8 (30%)	18 (67%)

**FIGURE 1:**
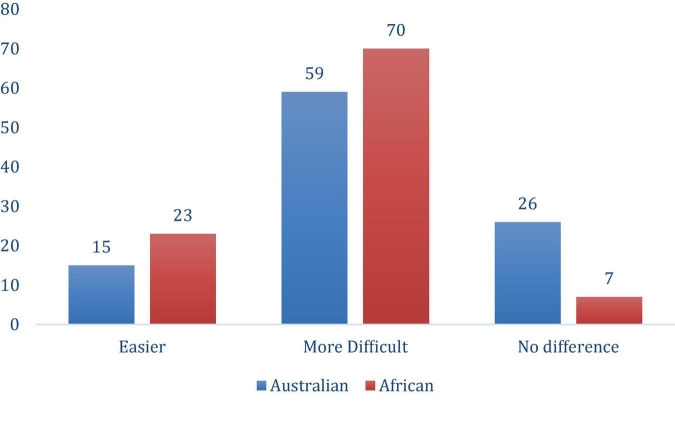
Experiences in managing workload.

In the Australian cohort of academics, most (59%), found it more difficult managing their workload followed by no difference (26%) in the pandemic period with the lowest response (15%) that it was easier to manage workload (see Figure 1).

A large majority (70.4%) of the African cohort found it more difficult to manage their workload since the start of the pandemic, especially with balancing both work and home duties (see Figure 1).

### Challenges faced, as a female academic, since the onset of the pandemic

An open-ended question in the survey asked respondents to comment on what they perceived were the greatest challenges they faced with their workload, as a female academic, since the pandemic started. As reported in Table 3, the Australian female cohort rated work–life balance as the number one theme (59%) followed by health and wellbeing challenges (28%) and a perceived lack of support by their higher education institution leadership (21%). Other lower rated themes included research related challenges, gender disparities, and managing online teaching. From the top theme, comments such as the following were reported highlighting the challenges of work–life balance (WLB): *“Trying to work from home with a 2yr was an absolute disaster – had to work while he napped and at night after he went to bed (Respondent 5)”; “Managing family and care needs of family whilst juggling never ending virtual online meetings at all hours (Respondent 8).”*; “*A work–life balance is really important for health and wellbeing but we are pushed and pushed to do more. This is not okay, especially when we are women and also raising children and looking after household duties. We are not superwomen although we are expected to be (Respondent 70).”*

**TABLE 3:** Australian challenges.

Australian challenges	Number	(%)
Work–life balance (WLB)	85	59
Health/wellbeing	40	28
Lack of support (HE Leadership)	30	21
Research	20	14
Gender	17	12
Online teaching	14	10

Health issues were also stated as follows:

*“health is in an issue and I prefer to work at home also have elderly parents in the home and do not want to expose them- this is seen as weak and not committed. Perhaps a male with similar problems would be given greater slack. In females health and home and carer issues are minimised (Respondent 30).”; “My children have struggled with mental health (Respondent 70).”*

Lack of support was stated as follows:

*“Coping with initial heavy workload, no support and subsequent job loss due to redundancy after 21 years (Respondent 130).”*

Research has been impacted due to the pandemic and this has also caused challenges for female academics as stated as follows: *“Inability to conduct fieldwork (etc) overseas, which is a major part of my research needs (Respondent 35)” and gender issues were also raised in perceived inequities in research opportunities: “Males used lockdowns and online teaching to write papers, while females took on extra admin/engagement, domestic and carer roles (Respondent 104).”*

In the “Gender” theme, there were many statements about perceived lack of care for female academics: *“No one cares about what is happening to women academics (Respondent 121).”; “females identify challenges of procedures and process and are identified as posturing for power; the rhetoric in online zoom meetings are generally male voices in meetings and in the decision making process (even if females have more experience and expertise in the decision-making area (Respondent 115).”*

Another major challenge has been identified as the theme of “Online teaching”:

“Lack of resources/tight budgetary constraints and no adequate training in online education are my main concerns.ne teaching *(Respondent 33”) and “Zoom fatigue. Being more exhausted as I am doing household work and work at the same time (Respondent 140).”*

While 14 themes emerged from the open-ended question on personal challenges which the African cohort faced since the onset of the pandemic, as summarized in Table 4, the top-3 personal challenges faced by the African cohort of female academics were WLB (56%), Extended working hours (15%), and access to a private workspace (15%). Four (4) respondents (15%) indicated that they faced no personal challenges since the onset of the pandemic.

**TABLE 4:** African challenges.

African challenges	Number	%
WLB	15	56
Extended working hours	4	15
None	4	15
Private workspace	4	15
Institutional resources	4	15
Social isolation	4	15
Health issues	2	7
Lack of support	2	7
Less opportunities	2	7
Uncomfortable with online	2	7
Burnout	1	4
Economic difficulty	1	4
Fear and uncertainty	1	4
Lack of professionalism	1	4

A complete list of emerging themes on personal challenges faced are presented in Table 4.

Work–life balance was difficult to maintain as some academics did not have access to a domestic assistant. Respondent 143 stated “*I no longer have domestic help (it is not safe health wise for either of us) so I am doing more domestic work. I have a capable husband who shares the load though. He does all the cooking, so all in all it is an equal load, with extra work for both of us.”* Respondents 9 and 143 added that both “*Family responsibilities and Employment duties”* increased. This was supported by Respondent 28, who highlighted that “*At work they forget that if you work from home you are juggling.*” Respondent 31 also stated that she faced a “*Struggle between work tasks and house chores.” “*…*it is also difficult to completely ignore undone household chores during “work hours” which results in tension within the household”* added Respondent 161. The absence of *“time for self”* (Respondent 182), resulted in *burnout* for some.

*“Long working hours”* (Respondent 35) was cited by a number of respondents. Knowing when it was *“time to stop working”* and end *“meetings and discussions”* (Respondent 260) was difficult. Respondent 145 stated that she found that *“work responsibilities take longer in an online way,”* forcing you to work beyond normal hours of duty, *“I cannot work around the clock and yet the university management seems to expect it,”* added Respondent 161.

Not all academics were blessed with a home office setup as the forced transition to work from home was made in the early stages of the pandemic. Comments related to the absence of a conducive *physical workspace* highlighted this issue, e.g., *“Working from home, with a new baby around”* (Respondent 45). Respondent 258 added, *“the consciousness of the four walls of the workspace”* with *“distractions”* becomes more difficult when *“it is difficult to ignore family members when they want to talk, especially as I do not have a separate office space to use”* (Respondent 161).

Access to *institutional resources* also posed a challenge, as Respondent 258 outlined *“inability to physically access reading materials from the library.”* Respondent 161 shared how this impacted on her own research, stating that *“a research project had to be put on hold as it required students to engage in laboratory-based practical activities throughout 2020 and 2021, and these were completely suspended due to the move to online teaching initially, and then the practical difficulties with implementing COVID protocols with limited space/equipment and large classes.”* Respondent 260 shared her challenge of *“accessing students work on all platforms.”*

*“The lack of face-to-face contact with colleagues has resulted in me feeling isolated*… *their isn’t anyone to share experiences and concerns with or engage in regular discussions with my colleagues around issues pertaining to teaching/learning/research,”* Respondent 161, highlighted the social isolation academics faced. Respondent 261 supported this, adding, *“I miss the stimulation and encouragement of work colleagues. I feel isolated and not sure what is happening in the academic world.”* Even non-work-related social isolation was cited, *“the ongoing lockdowns and being unable to see family have been challenging”* (Respondent 188).

*“Health deterioration”* (Respondent 63) and *“COVID sickness”* (Respondent 182) contributed to *health issues* faced.

Respondent 45 indicated that “*finding support/mentors*…” in particular “*finding the right support for where I am at,”* was challenging. In Africa, in particular, the lack of mentorship for females is more prevalent “…*hard to get men or women to really mentor other women*” (Respondent 15). She further highlighted that in Africa, there are *less opportunities “for women to get real practical IS skills”*…*“most organisations want men”*… *“Culturally, women have other duties to do. housework, childcare etc.”*

Respondent 161 indicated that she was *uncomfortable with online teaching* as she found it *“alien and disconnected.”*

*“Significant increase of cost of living”* was highlighted by Respondent 63. In addition, Respondent 161 added she was gripped with *fear and uncertainty*, *“I have felt emotionally stressed and drained by the worry of Covid, and how it might affect my world which affects my work.”*

### Opportunities created, personally, and as a female academic, since the onset of the pandemic

Another open-ended question asked respondents to comment on what they perceived as the top3 “work-related opportunities,” referring to positive opportunities from the challenges of a pandemic, and what academics could take forward beyond the pandemic period. Slightly more than 50% of respondents stated “online” opportunities as the top theme followed by teaching (31%) and flexibility (26%). A complete list of emerging themes on work-related opportunities perceived by Australian academics are presented in Table 5.

**TABLE 5:** Australian work-related opportunities.

Australian work-related opportunities	Number	%
Online	75	52
Teaching/Learning/Assessment	45	31
Flexibility	37	26
Nil/None	31	22
Conferences/PD	26	18
Time efficiency	23	16
Collaboration	23	16
Research	20	14
Time for self/others	12	8
Leadership role	8	6
Innovation	5	3
Other	4	2

A number of online work-related opportunities were realized during the pandemic such as ease of remote work and meetings, ability to develop new relationships, and increased online education and related skills as reported by respondents:

*“Virtual consultations (multi-campus institution), relationship building, up skilling in online education”* (Respondent 111); *“Opportunities to upskill in delivery of online content”* (Respondent 61).

The online theme extended into the teaching theme:*“Learning how to use different technologies (Zoom/Teams, Slido*, …*)”* (Respondent 13);*“Ability to develop online teaching practices”* (Respondent 136) and *“learning how to manage the transition of in person teaching mode to fully online”* (Respondent 77).

Flexibility was mentioned as a key opportunity allowing the academics to manage work and home giving them a greater WLB:

*“easier to manage family care responsibilities; greater work–life balance.”* (Respondent 8); *“more flexibility in working hours, no commuting”* (Respondent 29).

An interesting response by just over 20 percent of respondents was that they could see no opportunities coming from the pandemic:

*“If asking for opportunities for positive change in some way – none, I was drowning before and am drowning much more now”* (Respondent 104); *“None, the university has less need for my skills. My workload as a sessional tutor has decreased by 75%”* (Respondent 32); *Do you mean good things that have come of this? NONE”* (Respondent 58)

As presented in Table 6, not all was gloomy in Africa during the pandemic. Respondent 47 shared that in the *more relaxed atmosphere*, she *“felt more content*…*no stress of needing to present an appearance of being together.”* Respondent 143 stated that she was engaged in *self-reflection* during *“the pandemic and ongoing lockdowns*… *given me a lot more time, and reflective, thinking opportunities.”* Respondent 146 noticed that there was an opportunity for new student types during the pandemic stating a *“growth in student number and diversity for postgraduate degrees – mostly working students who enjoy the flexibility of online learning.”* While many academics battled with *student engagement* in the fully online environment, Respondent 260 enjoyed better engagement citing that *“Class discussions are great.”*

**TABLE 6:** African work-related opportunities.

African work-related opportunities	Number	%
Flexibility	13	48
Virtual collaboration	7	26
Virtual networking	6	22
Professional development	5	19
New technologies	5	19
Research time	3	11
Multitask	2	7
Funding	2	7
Family time	2	7
None	2	4
Self-reflection	1	4
New types of students	1	4
Economical	1	4
Access to research	1	4
More relaxed atmosphere	1	4

Notably the greatest opportunity came from the *flexibility* which the new work setup brought with it. Respondent 33 enjoyed *“teaching from home,”* which as Respondent 258 added allowed for *“increased flexible tutoring.”* The flexibility also allowed some academics to *“dedicate additional time to students,”* Respondent 9, with uninterrupted *“chunks of time,”* Respondent 27, as well as *“flexibility to attend to family duties,”* Respondent 9. The ability to reach a wider spectrum of students was highlighted by Respondent 31, *“more teaching opportunities, not controlled by geographic proximity-online.”* Flexibility with personal time was also indicated – *“some flexibility with time …am able to attend webinars at all hours,”* Respondent 161.

Increased *virtual networking and virtual collaboration* were highlighted by a number of respondents. *“One of the ironic and unexpected consequences of the pandemic is that fellow academics and researchers all over the world are now much more comfortable communicating in online formats. This has made international collaboration much easier. I have also been able to participate in global events without physically traveling which is a real blessing on the whole. I have met and worked with fellow researchers; we have developed such strong connections that I completely forget that we have never actually met face-to-face”* stated Respondent 143. This was supported by Respondent 188, who stated, *“I feel that I have had more opportunity to pursue my research career*…*I feel this is due to the ability to attend global conferences virtually.”* Respondent 31 added that she experienced an *“increase(d) interaction with academics across borders*…” thus creating *“more research opportunities with people from all over the world”* (Respondent 146). An increase in *“Faculty research collaboration”* was noted by Respondent 8, with more *“opportunities for sharing and peer discussions”* (Respondent 63).

Opportunities for *Professional Development* were created in *“online learning, teaching & assessment*… *being able to attend free webinars/conferences online that are presented all over the world that either did not exist previously or I was not able to access previously due to lack of funds to travel”* (Respondent 161). This was supported by Respondents 182, 260, and 261 *“attending online conferences I would not be able to attend easily as a single parent.”*

Respondent 35 spoke about the ability to experiment with *new technologies* like *“RPA”* in online teaching. Respondent 146 highlighted the *“growth in innovative approaches to teaching using online tools like Mural, Mentimeter, Kahoot, etc.”* Even in the Arts there was the introduction of new technologies in *“online programmes for teaching music education,”* Respondent 149.

Respondent 63 alluded to having *“more time for research when working from home.”* This was supported by Respondents 79 and 148.

Respondent 35 supported the notion of working from home stating, *“Let’s stay at home. It is better to be a good parent as you can multitask.”*

*Funding “for online research”* (Respondent 258) and *“research grants”* (Respondent 25), increased during the pandemic.

Respondent 9 indicated that working at home gave her the *“flexibility to attend family duties and”* allowed her to give *“quality time to children.”* This was supported by Respondent 28 who stated that she appreciated *“spending more time with family*… *and I actually got time to see my daughter grow and was there for each milestone.”*

*“Working online is more economical”* according to Respondent 9.

Respondent 15 felt that she had *“access to more online journals”* during this period.

### Supervisor responsiveness to the needs of a female academic work–life balance

As can be seen in Table 7, the highest selected response for both the Australian and African respondents was that they felt their immediate supervisor was responsive to their needs as female academics. The lowest response was to the “not at all” category. Overall, (83%) of Australian based academics and (89%) of the African based academics, stated that their supervisor was responsive to their work–life balance needs (see Table 7).

**TABLE 7:** Supervisor responsiveness.

	My immediate supervisor is responsive to my needs as a female academic (work–life balance):				
	Yes, all of the time	Most of the time	Sometimes	Not at all	Total
**Australia**	52 (36%)	38 (26%)	30 (21%)	24 (17%)	**144 (100%)**
**African**	7 (26%)	9 (33%)	8 (30%)	3 (11%)	**27 (100%)**

## Discussion

The result from this research supports the previous work that the female academics face challenges, which need to be considered by policy makers in institutions (Morley et al., 2006). However, this comparative study clearly demonstrates that although challenges are present, geographic locations have brought different challenges within the context of teaching in a HEI.

The result clearly demonstrates the work from home was perceived very positively by female academics both in Australian and Africa, with both cohorts reporting the ease of managing working from home with others; children, partners, parents, friends, or co-residents. Although this was a positive outcome, further analysis on managing workload show the need for support for the female academics. Supporting this premise is that seven out of 10 African female academics found it more difficult to manage their workload since the start of the pandemic, especially with balancing both work and home duties compared to just 6 out 10 Australian female academics. In terms of challenges faced by the cohort, there were distinct differences between the cohort. The Australian female cohort reported the top-3 challenges to be work–life balance, health and wellbeing and the perceived lack of support by their higher education institution leadership. The African cohort’s top-3 challenges were WLB, extended working hours, and access to a private workspace. This finding also supports the research work of Ravi and Anulakshmi (2021) and Mani (2013) that work–life balance of women employees is an integral issue in employment but is specific to regional settings (Singh et al., 2022). This study further shows that both the Australian and African respondents felt their immediate supervisor was responsive to their needs as female academics. The study suggests that there has been development of support mechanisms during the pandemic for female academics during the pandemic environment. However, even with this advancement of support for their institutions the study suggests that during the pandemic, workloads were an issue and impinged on the work–life balance, suggesting that the leadership in higher education institutions develop policies and procedures to support this balance. An approach would include female academics in the process for change at the institutional level.

Though challenges were prevalent, this study also showed that during the pandemic setting there were also work-related opportunities. The Australian cohort listed their three top opportunities as “online” opportunities which included remote work and meetings, ability to develop new relationships and increased online education and related skills; teaching; and flexibility. The African cohort on the other hand listed flexibility; virtual collaboration; and virtual networking as the top-3 opportunities. Both cohorts had similar perceptions that the online opportunities and flexibility was critical positives in the pandemic setting.

## Limitations

There were a number of limitations to this current study including this being a small sample of a large cohort of female academics’ experiences during COVID-19 across the globe. In particular, the African cohort was much smaller than the Australian sample, despite numerous attempts to reach out to female academics in Africa to participate in the study. There were differences in the Australian and African isolation and lockdown times, rules, and freedoms afforded in both locations which is acknowledged as a limitation of the study. Another limitation, due to COVID was the inability to run any focus groups in person and the organization of online formats would have been difficult to arrange within given timelines at the time of preparing research articles.

The future research looking at policies and practices within Australia and Africa will shed further light on female academics’ experiences during this period.

## Conclusion

This study adds to the academic literature of female academics on their work–life balance during the COVID-19 pandemic. Effects such as increased workloads, additional domestic work, and extended works have impacted on the health and wellbeing among female academics.

This research compares the opportunities and challenges faced, as well as the support received, by female academics in Australia and Africa. The study shows that the geographic locations of the female cohort results is differing support needs suggesting that policy makers have to be aware of the specificity of the cohort. Interestingly, this comparative study also highlighted that notwithstanding the geographic locations, female academics were on the same page in terms of work-related opportunities, primarily the online learning opportunities, and flexibility in the pandemic setting.

## Data availability statement

The original contributions presented in this study are included in the article/supplementary material, further inquiries can be directed to the corresponding author.

## Ethics statement

The studies involving human participants were reviewed and approved by University of Western Australia. The patients/participants provided their written informed consent to participate in this study.

## Author contributions

All authors listed have made a substantial, direct, and intellectual contribution to the work, and approved it for publication.
